# A Novel Platform of MOF for Sonodynamic Therapy Advanced Therapies

**DOI:** 10.3390/pharmaceutics15082071

**Published:** 2023-08-01

**Authors:** Donghui Liao, Jiefeng Huang, Chenyi Jiang, Luyi Zhou, Mingbin Zheng, Alireza Nezamzadeh-Ejhieh, Na Qi, Chengyu Lu, Jianqiang Liu

**Affiliations:** 1 Guangdong Provincial Key Laboratory of Research and Development of Natural Drugs, School of Pharmacy, Guangdong Medical University Key Laboratory of Research and Development of New Medical Materials, Guangdong Medical University, Dongguan 523808, China; liaodonghui0017@163.com (D.L.); hjf791616775@126.com (J.H.);; 2 Chemistry Department, Shahreza Branch, Islamic Azad University, Shahreza 311-86145, Iran; 3 Key Laboratory of Biorheological Science and Technology, Ministry of Education, College of Bioengineering, Chongqing University, Chongqing 400030, China; 4 Affiliated Hospital of Guangdong Medical University, Zhanjiang 524013, China

**Keywords:** metal–organic frameworks, sonodynamic therapy, sonosensitizer

## Abstract

Metal–organic frameworks (MOFs) combined with sonodynamic therapy (SDT) have been introduced as a new and efficient treatment method. The critical advantage of SDT is its ability to penetrate deep tissues and concentrate energy on the tumor site to achieve a non-invasive or minimally invasive effect. Using a sonosensitizer to generate reactive oxygen species (ROS) under ultrasound is the primary SDT-related method of killing tumor cells. In the presence of a sonosensitizer, SDT exhibits a more lethal effect on tumors. The fast development of micro/nanotechnology has effectively improved the efficiency of SDT, and MOFs have been broadly evaluated in SDT due to their easy synthesis, easy surface functionalization, high porosity, and high biocompatibility. This article reviews the main mechanism of action of sonodynamic therapy in cancer treatment, and also reviews the applications of MOFs in recent years. The application of MOFs in sonodynamic therapy can effectively improve the targeting ability of SDT and the conversion ability of reactive oxygen species, thus improving their killing ability on cancer cells. This provides new ideas for the application of micro/nano particles in SDT and cancer therapy.

## 1. Introduction

A tumor is an abnormal pathological change caused by the abnormal clonal proliferation of a cell that loses its normal growth regulation at the gene level in various tissues due to various carcinogenic factors’ action. The existence of cancer seriously affects people’s quality of life. In recent years, many new cancers and their resulting deaths have been observed [1,2]. Traditional therapeutic methods can be subdivided into three types: surgery, systemic chemotherapy, and radiation therapy, which are still the main clinical methods for treating tumors, with certain positive effects. However, the side effects are obvious, and their treatment efficiency is low. It is a matter of great practical significance to introduce/develop safe and efficient therapeutic methods [3,4,5,6].

In response to the shortcomings of traditional treatments, more and more new therapies are being proposed, including chemodynamic therapy (CDT), photodynamic therapy (PDT), and SDT, which will slowly become alternative therapies to traditional treatments in terms of development trends. These new therapies generate large amounts of ROS in the tumor microenvironment to kill tumor cells. ROS include hydroxyl radical (·OH), singlet oxygen (^1^O_2_), superoxide (O_2_^−^), and peroxide (O_2_^2−^) [7,8].

ROS function as a double-edged sword in cells [9]. The production of ROS has long been considered one of the key factors in protecting our bodies via disease resistance, cell-mediated immunity, and microbicidal activity by acting as a cellular signal. The cells themselves have multiple regulatory mechanisms to maintain the stability of ROS levels; low levels of ROS maintain cell cycles and participate in their proliferation and protein synthesis [10]. However, when the balance between ROS generation and elimination is broken, it is easy to produce high levels of ROS that trigger an oxidative stress response. This leads to oxidative damage to cellular components, apoptosis, or necrosis and may promote carcinogenic mutations [11,12]. Therefore, oxidative stress caused by high ROS levels can also be applied in cancer cell therapy [13]. At the same time, many studies have found that ROS levels in tumor cells are generally higher than in normal cells, and cancer cells increase the expression of endogenous antioxidants (e.g., glutathione or catalase) to adapt to this redox state. This flexible redox stress response makes cancer cells more resistant to exogenous stress responses such as surgical treatment, radiotherapy, and chemotherapy [14,15,16,17,18,19,20,21], creating a barrier to traditional treatments. Cancer cells maintain their own stability by increasing the production of endogenous antioxidants, which causes cancer cells to have strong instability in the production of exogenous ROS, which will be one of the research directions for tumor therapy in the future [22].

Unlike SDT, PDT was studied as early as the 19th century. The first part of PDT treatment is photosensitizers—a light-sensitive molecule that can be targeted at cancer cells or tissue. In the second part, after activating the photosensitizer with a specific light wavelength, the transferred energy from the photosensitizer to molecular oxygen produces ROS. In other words, this sequence of reactions can only occur at specific sites where the photosensitizer is present [23]. Moreover, ROS is characterized by high activity and a short half-life (<0.04 μs), so that it can only produce effects near the production site (<0.02 μm) [24]. The main clinical limitation of PDT is its penetration depth, resulting in poor therapeutic effects on deep tissues and organs. The treatment effect is better for superficial tumors, such as skin cancer [25]. Although near-infrared (NIR) light has been utilized to improve the therapeutic depth of photodynamic therapy, the loss of energy in the irradiation process and the effect of the skin on its penetration ability is still a direction to be investigated [26].

**Figure 1:**
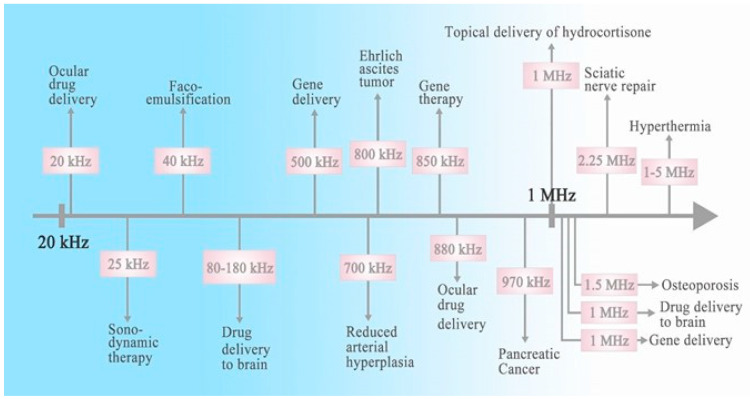
A summary of biomedical applications of different frequencies of ultrasound.

SDT was developed based on PDT. The SDT treatment advantage relative to PDT is that it can penetrate deeper into tissues and organs without radiation, and, with a lower tissue attenuation factor, it can be tightly focused and penetrate tens of centimeters of tissue depending on the frequency applied [27]. SDT is a form of US therapy; US is defined as sound in the audible range of 20 kHz to 1 GHz, which generates longitudinal waves in fluids and gases and which is a basic acoustic energy form with a wide range of therapeutic and diagnostic uses [28]. Under the action of sound waves, different tissues will exhibit other propagation characteristics. This phenomenon is used in diagnosis via ultrasound-based imaging. Some attributes of US imaging are real-time observation, noninvasiveness, high efficiency and safety, and low cost [29,30]. In addition to classical diagnostic imaging, US is also used to promote drug release and transdermal drug delivery [31]. General biomedical applications of ultrasound at different frequencies are summarized in Figure 1.

Umemura et al. [32] explored the cell damage mechanism caused by ultrasound combined with hematoporphyrin after first proposing the concept of SDT at the International Ultrasound Conference in 1989. In the presence and absence of hematoporphyrin, mouse sarcoma were examined via ultrasound for 60 s, and active oxygen scavenger agents such as histidine and mannitol were administered. Based on these results, histidine inhibits the damage of hematoporphyrin to cells, while mannitol has no inhibiting effect. Histidine is a scavenger of hydroxyl radicals and singlet oxygen, while mannitol is a scavenger of hydroxyl radicals. The results showed that the ultrasound might have induced the hematoporphyrin to produce singlet oxygen and enhance cell damage [32]. In the application of SDT, ultrasound is usually combined with sonosensitizers. The unique mechanisms of ultrasound enable it to activate sonosensitizers in their ground state. When the excited-state sonosensitizers return to the ground state, they release energy and transfer it to the surrounding oxygen molecules, converting oxygen molecules into free radicals to produce many ROS. Combining sonosensitizers and ultrasound would be an effective method for inhibiting tumor growth while reducing potential drug resistance and the limitations of a single treatment method. Photosensitizers served as the basis for the first generation of sonosensitizers. The earliest sonosensitizers involved the use of photosensitizers such hematoporphyrin or porphyrin derivatives, which are not poisonous by themselves, show anti-tumor effects in vivo after being activated via ultrasound, and have high sound sensitivity and catalytic ability. Some chemotherapy drugs, such as doxorubicin (DOX) and cytarabine, also have the effect of sonosensitizers. However, common organic sonosensitizers frequently come with drawbacks such as high hydrophobicity, poor chemical/biological stability, and a lack of tumor selectivity [33,34,35].

In comparison, inorganic sonosensitizers, such as TiO_2_, although non-toxic to animal cells, chemically inert, and stable under physiological conditions, also suffer from poor biodegradability, biocompatibility, and relatively low ROS quantum yield. There are still relatively few studies using TiO_2_ in combination with SDT for cancer therapy [36,37]. The major feature of hypoxia in the tumor microenvironment is also one of the reasons for the low efficiency of SDT. Therefore, developing satisfactory sound-sensitive agents is significant in increasing SDT efficiency and clinical application. To overcome these problems, researchers have begun to encapsulate sonosensitizers into micro/nanocarriers or design drugs to assemble into nano systems. On the one hand, direct physical packaging of sonosensitizers can effectively protect the function of sonosensitizers and improve the efficacy of SDT when sonosensitizers reach the tumor microenvironment. On the other hand, a nano platform composed of organic sonosensitizers and micro/nanomaterials can be modified to grant it higher biocompatibility or tumor targeting ability and improve the efficiency of SDT. Therefore, combining micro/nanomaterials with sonosensitizers for SDT is of great research value.

**Figure 2:**
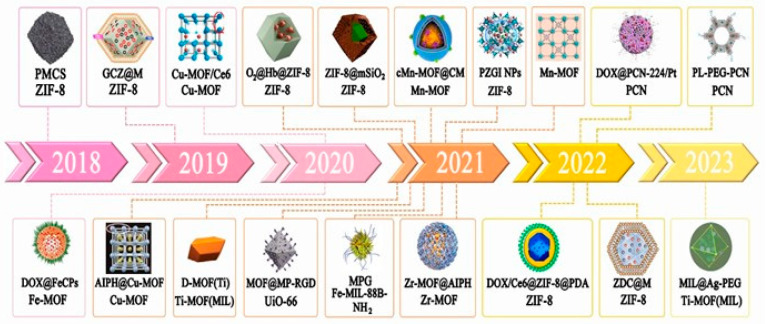
Application of MOFs in SDT in recent years.

MOFs, or porous coordination polymers (PCPs), are based on metal ions as the connection point bridging organic–inorganic hybrid nanomaterials’ porous three-dimensional mesh nano-framework material. Due to their special structure, they have a wide variety of applications including multiphase catalysis, gas adsorption, chemical sensing fields etc. [38,39,40,41,42,43,44,45,46,47,48,49,50,51,52,53,54,55,56,57,58,59]. Recently, MOFs have been extensively developed in the medical field, especially in cancer treatment. MOFs have the following advantages: First, due to their ease of synthesis, MOFs materials with simple surface modifications can be synthesized from different proportions of metals and ligands while maintaining uniformity and shape control. Second, their large surface area and high porosity enable MOFs to have high drug-loading capacity. Third, MOFs have high biodegradability and certain targeting due to combining organic–inorganic hybrid materials. By adjusting the ratio of organic–inorganic hybrids, many different MOF structures can be constructed, better tailored to various complex environments, and widely used in many fields [60,61,62,63,64,65,66,67,68,69,70,71,72,73,74,75,76,77]. Traditional sonosensitizers lack stable targeting ability and stability in the treatment process and are prone to decomposition in the complex human microenvironment. In numerous reports, the addition of drug carriers or combination with other therapeutic methods can effectively improve the therapeutic effect of SDT. Therefore, the application of MOFs in SDT can effectively solve the existing problems in SDT. It is of great significance to use MOFs to create SDT treatment platform for cancer treatment, which provides new ideas for the development of SDT. The size of MOFs plays an important role in the efficacy of SDT. Therefore, in the process of research, emphasis should be placed on the synthesis of MOFs of appropriate size for the construction of therapeutic platforms. At the same time, the metabolic process of MOFs in vivo, the understanding of which is lacking in many studies at present, also needs to be clarified. This article reviews the mechanism of action of sonodynamic therapy in cancer treatment and the application of MOFs in SDT in recent years and puts forward future prospects and challenges based on the existing research results. The application of MOFs in SDT in recent years is shown in Table 1 and Figure 2.

There is no exact conclusion on the mechanism of ultrasound, which is relatively complex. The most direct US effect is raised temperatures, followed by the cavitation effect, that is, the growth and rupture of bubbles, also including the continuous oscillation of bubbles; the former is called inertial cavitation, the latter stable cavitation. Both can induce critical physical, chemical, and biological effects. It should also be noted that US waves induce thermal and non-thermal effects. The stable cavitation effect causes bubbles to oscillate continuously, and the liquid around the bubbles accelerates the flow, causing continuous contact and collision with surrounding materials. In inertial cavitation, the bubbles keep becoming bigger and collapsing. The temperature at the core of the bubble can reach temperatures of more than 1000 K, and the shock wave caused by the collapse has an amplitude of over 10,000 atmospheres, depending on the bubble’s size. Therefore, the degree of influence of ultrasound on tissue critically depends on the frequency and amplitude of the ultrasound. The thermal effect is the conversion of mechanical energy into thermal energy during the ultrasound process, which is induced in tumors via high-intensity focused ultrasound (HIFU). The heat generated during this process raises the temperature of the ultrasound site. This acts on tumor cells and causes changes in cell membrane fluidity, which is one of the reasons to promote transdermal drug delivery. In addition to cavitation, the pyrolytic effect and sonoluminescence (SL) can also result in ROS generation. The local temperature rise caused by the pyrolysis effect of ultrasound can also cause the cracking of sonosensitizers and the production of free radicals. SL generates light in solutions under ultrasonic irradiation and converts sound energy into heat and light, which may be a medium for ultrasound-activated sonosensitizers [78,79,80,81,82,83,84,85,86]. In the study by Sazgarnia et al., protoporphyrin IX (PpIX)-coupled Au nanoparticles (Au NPs) were used to successfully demonstrate the sonoluminescence of gel-based phantoms under ultrasonic irradiation at a frequency of 1.1 MHz.

**Figure 3:**
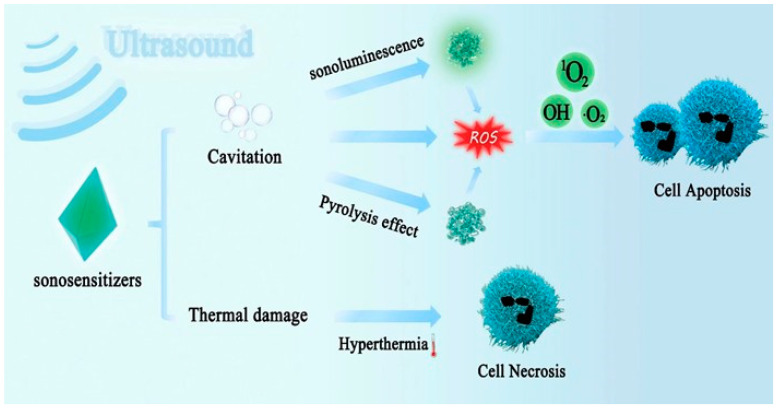
The scheme of possible mechanisms of SDT.

In the same investigation, sonoluminescent signals were found at 350 to 450 nm, 450 to 550 nm, and 550 to 650 nm, in which Au NPs acted as the nodes of cavitation nuclei [87]. The possible SDT mechanisms are shown in Figure 3. The therapeutic, HIFU, and diagnostic ultrasound frequency ranges were 3 to 30 MHz, 1 to 3 MHz, and 20 kHz to 3 MHz, respectively. The exact ultrasonic frequency is usually not specified and would be adjusted according to the actual situation during use [88]. Typically, the SDT process is administered via low-intensity ultrasound in combination with drugs to induce tumor cell death. Ultrasound diagnosis causes minimal tissue damage, so the intensity of ultrasound diagnosis is lower than that of SDT and HIFU. The exact mechanism of SDT has not been fully explored, mainly the cavitation effect, ROS, and ultrasound-induced apoptosis. Still, studies have demonstrated that ultrasound activation of sonosensitizers to generate ROS plays a key role in inducing tumor cell death [80,89,90].

Low-intensity US alone is generally not used for cancer treatment. After cell damage, single low-intensity ultrasound treatment can cause repair mechanisms to stimulate tumor growth and increase the permeability of cell membranes and blood vessels, thereby promoting the oxygen supply to the microenvironment around a tumor, allowing tumor cells to grow. Zhao et al. [91] reported enhanced epirubicin hydrochloride (EPI) inhibition of tumor growth in an in vivo ultrasound study. The effect of US on tumor cell drug uptake was evaluated via phospholipid-based microbubbles (PMB) in combination with chemotherapy drug injection. The experiment was divided into a blank control group and five experimental groups. By comparing the relative growth rate of tumors under 5 consecutive treatments, it could be seen from the first group (US + PMB) that there was a downward trend in the first three days of treatment and an upward trend in the second two days; that is, US combined with PMB had a dual effect of short-term inhibition and long-term activation on tumor growth. It could be seen that the low-power ustic manipulation effect could trigger the repair mechanism after cell injury, which may lead to tumor growth if antitumor drugs are not used [91].

## 2. Application of MOFs in SDT

### 2.1. Application of MOFs as Sonosensitizer Carriers in SDT

**Figure 4:**
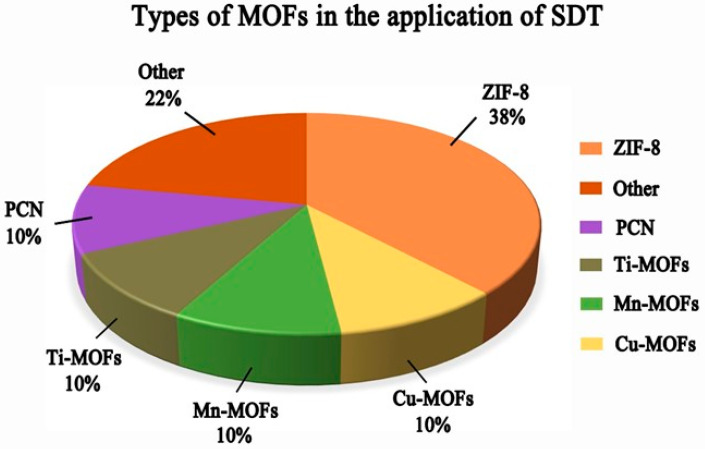
Types of MOFs in the application of SDT.

Biomedical MOF use has dramatically advanced in the recent ten years, particularly in the cancer therapy area [92,93,94,95]. Compared with common organic or inorganic materials, MOFs have a sizeable effective surface area and porosity and are easy to modify after synthesis. MOFs can transport drugs, sonosensitizers, photosensitizers, and antibodies to the targeted site, improving treatment efficiency and reducing side effects; types of MOFs in the application of SDT are shown in Figure 4. Among them, zeolite imidazole skeleton (ZIF-8), a novel porous material formed from zinc ions and 2-methylimidazole, has unique advantages, including high porosity, good tunable structure, tunable surface functionality, and inherent acidity-induced biodegradability. This unique metallic structure effectively protects the sonosensitizers from elimination or degradation during blood circulation, allowing the sonosensitizers to be cleaved at specific sites to release energy to generate ROS. ZIF8, therefore, has significant potential for use as a sonosensitizer in SDT. The innovative sonosensitizers for SDT developed by Meng Yuan’s team in 2021 have excellent ROS generation ability and biocompatibility. Researchers created a nano-platform called Hb@ZIF-8 (HZ) by combining hemoglobin (Hb) and ZIF8 via a one-pot method as an organometallic framework. Hb is a natural metalloporphyrin carrier and a natural O_2_ carrier, resulting in O_2_@Hb@ZIF-8 (OHZ) nanoparticles with excellent biocompatibility and a rich source of O_2_ for US-induced ROS generation.

**Figure 5:**
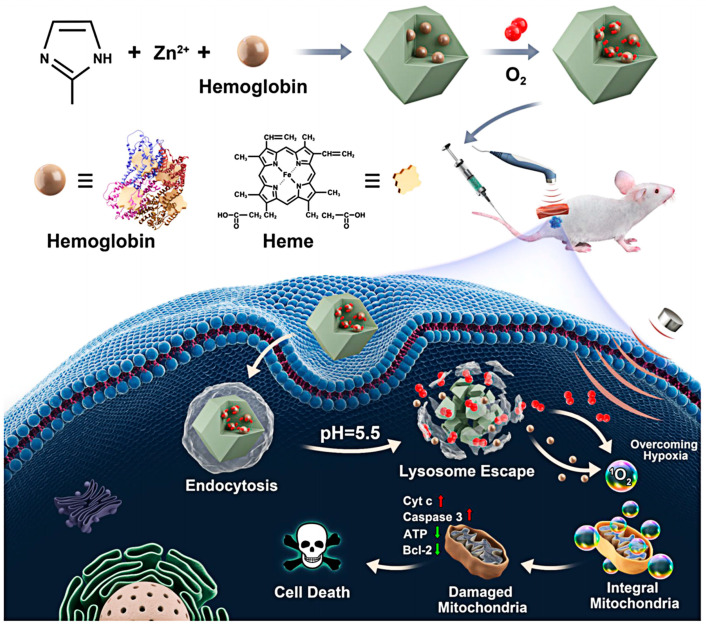
The OHZ NPs’ Main Synthesis Procedures and Antitumor Mechanisms. Reproduced with permission from ref. [96] (copyright 2021, Yuan).

**Figure 6:**
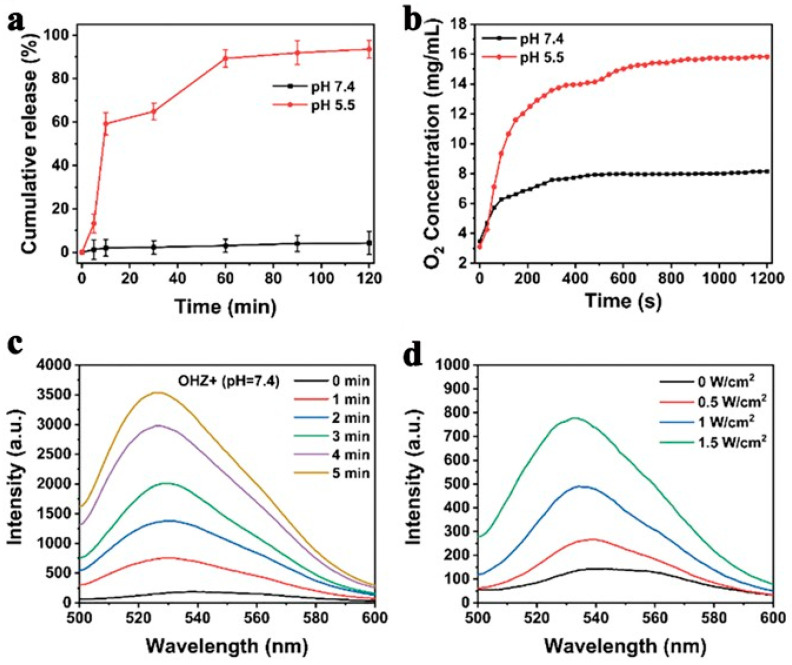
The effect of PH on release of (**a**) Hb and (**b**) O_2_ from OHZ. Time-dependent ^1^O_2_ produced from OHZ under US irradiation (1.0 MHz, 1.5 W cm^−2^, 50% duty cycle), using an SOSG probe. (**c**) Time-dependent ^1^O_2_ produced from OHZ under US irradiation (1.0 MHz, 1.5 W cm^−2^, 50% duty cycle), using an SOSG probe. (**d**) The intensities of fluorescence of SOSG coincubation with OHZ under various densities of US irradiation. Reproduced with permission from ref. [96] (copyright 2021, Yuan).

ZIF8, a stable acid-responsive degradation drug carrier, can effectively protect Hb from degradation before it enters the tumor microenvironment, increasing the effectiveness of SDT. Hb is released as a sonosensitizer in the tumor microenvironment and generates energy transfer to the surrounding media under ultrasound to produce ROS, which in turn causes severe mitochondrial dysfunction and activates the mitochondrial apoptosis pathway to kill tumor cells. The Hb and heme structures are presented in Figure 5. As shown in Figure 6a,b, in acid pH response experiments, the NPs maintained their structure in PBS at pH 7.4 for 1 h. The crystals degraded significantly at pH 5.5, and the highest cumulative release of Hb and O_2_ was observed, which could be related to the collapse of ZIF-8 at pH 5.5. This suggests that ZIF8 can effectively transport OHZ nanoparticles into the tumor microenvironment without disruption. ^1^O_2_ was detected via singlet oxygen sensor green (SOSG), and the fluorescence signal intensity enhanced with the increase of US power density and irradiation time (Figure 6c,d), indicating that US-stimulated OHZ NPs generated ^1^O_2_. To further explore the intracellular distribution of the nanoparticle platform, fluorescence co-localization of FITC and LysoTracker revealed that OH_FITC_Z or H_FITC_Z nanoparticles incubated with 4T1 cells for 4 h showed a clear green fluorescence signal in the cytoplasm, indicating the NPs had successfully escaped from the lysosome.

**Table 1:** Application of MOFs in SDT.

No.	Composition	MOFs	Synthesis Method	Grain Size	Treatment	Ref.
1	O_2_@Hb@ZIF-8	ZIF-8	One Pot Method	200 nm	4T1	[96]
2	DOX/Ce6@ZIF-8@PDA	ZIF-8	One Pot Method	135 nm	4T1	[97]
3	ZDC@M	ZIF-8	One Pot Method	240 nm	4T1	[98]
4	GSNO/Ce6@ZIF-8@Cytomembrane	ZIF-8	One Pot Method	179 nm	4T1	[99]
5	Zr-MOF@AIPH	Zr-MOF		~138 nm	PancO2	[100]
6	ZIF-8@mSiO_2_	ZIF-8	One Pot Method	180–190 nm	4T1	[101]
7	MIL@Ag-PEG	Ti-MOF (MIL)	Solvent heat method	230 nm	A549	[102]
8	PMCS	ZIF-8	One Pot Method	110 nm	4T1	[103]
9	cMn-MOF@CM	Mn-MOF	Solvent heat method	87.1 ± 1.0 nm	B16/H22	[104]
10	Mn-TCPP	Mn-MOF	Solvent heat method	70 nm	H22/4T1	[105]
11	DOX@FeCPs	Fe-HMME	self-assemble	~70 nm	4T1/CT26	[106]
12	PL-PEG-PCN	PCN-222	Solvent heat method	~185 nm	MCF-7	[107]
13	DOX@PCN-224/Pt	PCN-224	Solvent heat method	100 nm	CT16/SKOV3	[108]
14	AIPH@Cu-MOF	Cu-MOF		259.4 × 118.9 nm	Panc02	[109]
15	D-MOF(Ti)	Ti-MOF (MIL)	Solvent heat method	120 nm	4T1	[110]
16	MOF@MP-RGD	UiO-66	Solvent heat method	<200 nm	MDA-MB-231	[111]
17	MPG	Fe-MIL-88B-NH_2_	Solvent heat method	~200 nm	4T1	[112]
18	Cu-MOF/Ce6	Cu-MOF	One Pot Method	260 nm	MCF-7	[113]
19	Pt@ZIF-90@Gem@IR780	ZIF-90		~150 nm	BxPC-3	[114]

In contrast, almost no fluorescent signal was observed after 4 h of Hb_FITC_ action on 4T1 cells, which is due to the rapid breakdown of pH-responsive ZIF-8, leading to lysosomal swelling and rupture, which facilitates the escape of Hb from the lysosome. When the OHZ NPs’ biocompatibility was reached via the standard methyl thiazolyl tetrazolium (MTT) assay, a relative cell viability of 80% remained under both normoxic and hypoxic conditions for OHZ nanoparticle concentrations up to 100 μg/mL, which demonstrates the relatively moderate biocompatibility of OHZ NPs. The above results could indicate that ZIF-8 is an excellent MOF nanomaterial that can avoid the destruction of OHZ nanoparticles in the lysosome but can decompose in the microenvironment of a tumor to release Hb to generate large amounts of O_2_, alleviating tumor hypoxia while providing a rich source of O_2_ for US-induced ROS generation. This study makes the ZIF-8 carrier the best choice among more natural sonosensitizers. Hb has the features of high biocompatibility and low photosensitivity, making it a promising sonosensitizer for research, which provides the direction for developing safer and more efficient sonosensitizers [96].

**Figure 7:**
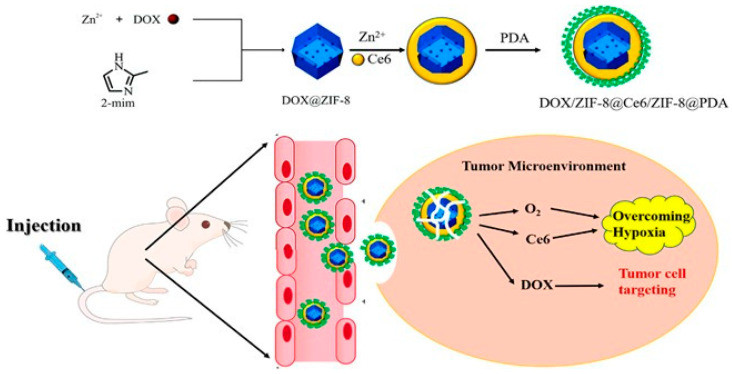
Construction of DOX/Ce6@ZIF-8@PDA therapeutic platform and schematic illustration of the action mechanism. Reproduced with permission from ref. [97] (copyright 2022, Zhong).

**Figure 8:**
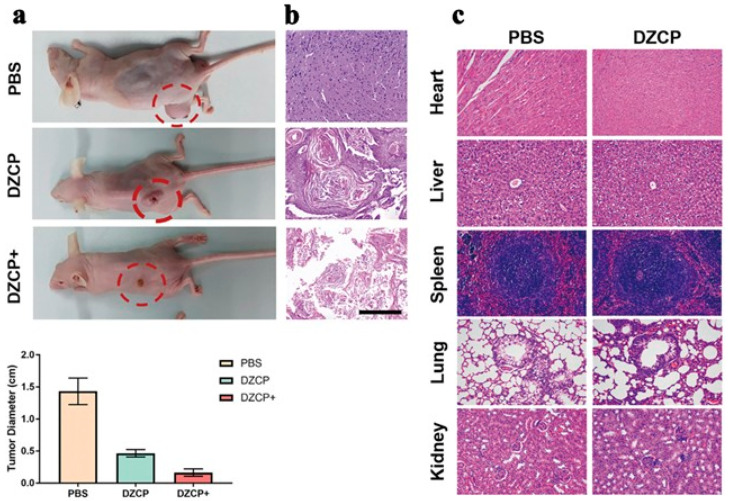
(**a**) 4T1 tumor-bearing mice at the end of treatments, comparing the PBS, DZCP, and DZCP groups. Histological pictures of treated (**b**) tumors and (**c**) different tissue sections. Scale bar is 50 μm. Reproduced with permission from ref. [97] (copyright 2022, Zhong).

In 2022, Zhong’s team used ZIF-8 with the sonosensitizer Ce6 and the anti-cancer drug doxorubicin (DOX) [97]. It was encapsulated with polydopamine (PDA) to finally obtain the DOX/Ce6@ZIF-8@PDA (DZCP) therapeutic platform (Figure 7) [97]. ZIF-8 is an excellent drug carrier that can deliver Ce6 and DOX simultaneously in this system to achieve synergistic SDT and chemotherapy effects. In addition, the selection of PDA to coat the platform promoted the endocytosis of the platform, thus enabling more precise drug delivery and increasing intracellular drug concentration to induce a higher lethality to cancer cells. In vitro drug release experiments, researchers studied the pH response drug release of the DZCP treatment platform, and the results showed that the drug release was negatively correlated with pH value. When the pH was reduced to 5.6, almost 90% of the Ce6 was released due to DZCP decomposition. When the pH was decreased to 5.6, roughly 88% of the DOX drugs were released. This indicates that ZIF-8 as a drug carrier has the advantages of good pH-acid response performance and low drug leakage. In the in vivo anti-tumor assay, researchers investigated the anti-tumor effect of DZCP in mice by intravenously injecting different drugs into 4T1 tumor-bearing Balb/c mice (Figure 8). The results showed that DZCP had a small tumor-suppressive effect in the absence of ultrasound, mainly due to the therapeutic effect of releasing the chemotherapeutic drug DOX. In contrast, under the effect of ultrasound, DZCP had a critically inhibitory effect on tumors in mice, which was mainly due to the synergistic therapeutic effect of SDT and DOX chemotherapy under the effect of ultrasound, thus enhancing the therapeutic effect. These experimental results all indicate that the DZCP therapeutic platform is an effective drug for tumor treatment and also show that ZIF-8 is a suitable drug carrier that can carry both the chemotherapeutic drug DOX and the sonosensitizer Ce6, thus achieving a synergistic therapeutic effect.

**Figure 9:**
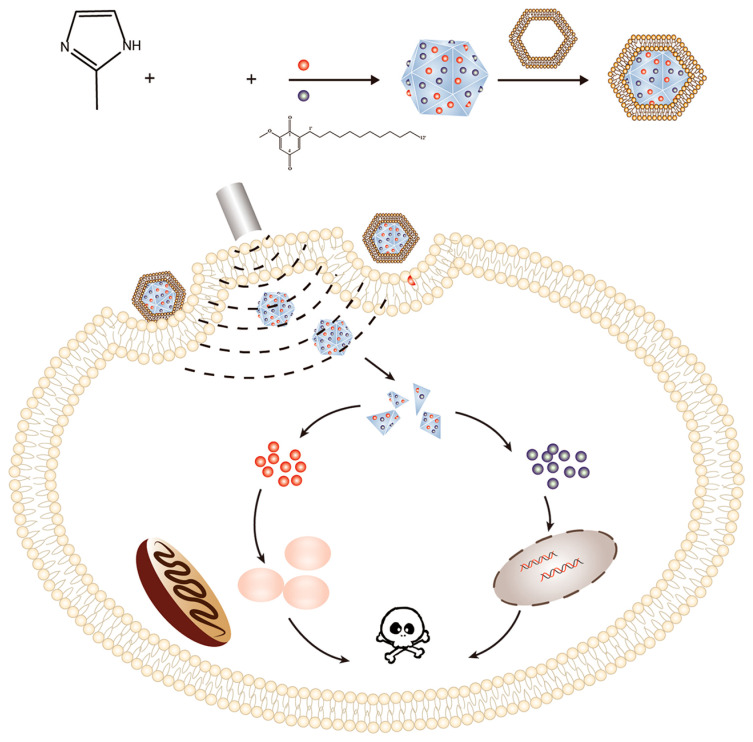
Schematic illustration of the synthesis process of the ZDC@M nanoparticles and pH-sensitive ultrasound-triggered SDT combined with DMDD therapy. ZDC@M, ZIF-8@ DMDD/Ce6@ cytomembrane; ZIF-8, zeolitic imidazole frameworks-8; SDT, sonodynamic therapy; DMDD, 2-dodecyl-6-methoxycyclohexa-2,5-diene-1,4-dione. Reproduced with permission from ref. [98] (copyright 2022, Zhao).

Similarly, in 2022, Zhao’s team used ZIF-8 as a carrier to carry the sonosensitizer Ce6 and 2-dodecyl-6-methoxycyclohexa-2,5-diene-1,4-dione (DMDD) extracted from Chinese herbal medicine [98]. Then it wrapped the drug-loaded system in a cell membrane of 4T1 cells to finally obtain ZIF-8@DMDD/Ce6@cytomembrane (ZDC@M) nanoparticles (Figure 9) [98]. In addition, the small diameter of ZDC@M nanomaterials, about 200 nm, allows the nanomedicine to easily penetrate the cell membrane and release the drug deep into the tumor cells, enhancing its ability to kill the tumor cells. In the in vivo antitumor experiments, the in vivo antitumor activity of ZDC@M nanomedicine was studied by establishing a nude mouse model carrying 4T1 cells. The results indicated that the inhibition of tumor growth in the ZDC@M + US group was significantly better than that in other experimental control groups, which was mainly because under the action of US, the sonosensitizer Ce6 was excited to generate ROS, which resulted in a synergistic effect with the antitumor drug DMDD, thus enhancing the performance of tumor growth inhibition. In 2019, An’s team then used ZIF-8 loaded with Ce6 to further encapsulate nitrosoglutathione (GSNO), which was wrapped in homologous tumor cell membranes, and ultrasound, which could trigger NO released from GSNO and ROS generated by Ce6 to cause damage to tumor cells with excellent biocompatibility and targeting ability [99].

### 2.2. Application of MOFs as Sonosensitizer in SDT

**Figure 10:**
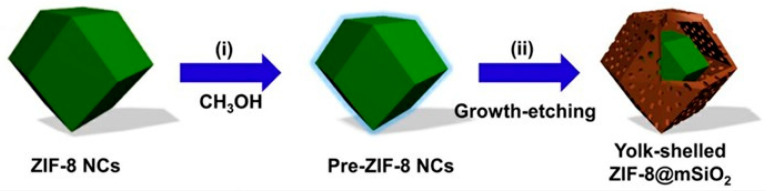
ZIF-8@mSiO_2_ synthesis. Reproduced with permission from ref. [101] (copyright 2021, Wang).

**Figure 11:**
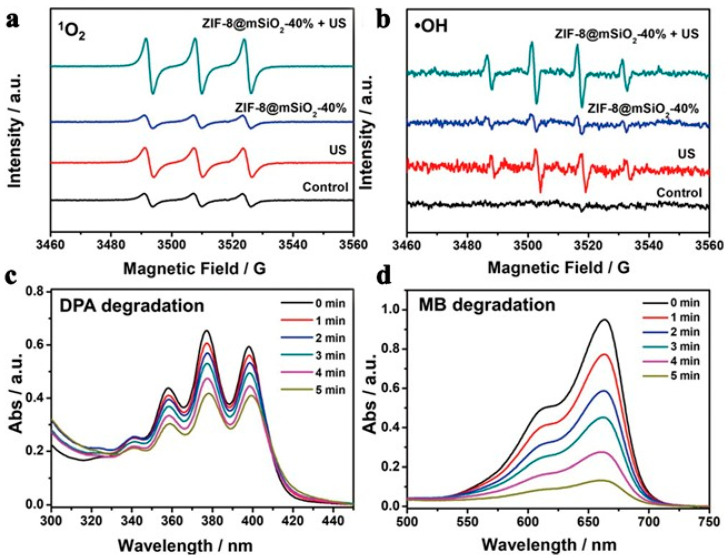
The yolk–shell ZIF-8@mSiO_2_ nanostructures sonodynamic feature. (**a**) ^1^O_2_ and (**b**)·OH production of yolk–shell ZIF-8@mSiO_2_-40% nanostructures (100 ug mL^−1^). (**c**) DPA degradation of yolk–shell ZIF-8@mSiO_2_-40% nanostructures (100 ug mL^−1^). (**d**) MB degradation of yolk–shell ZIF-8@mSiO_2_-40% nanostructures (100 ug mL^−1^). Reproduced with permission from ref. [101] (copyright 2021, Wang).

**Figure 12:**
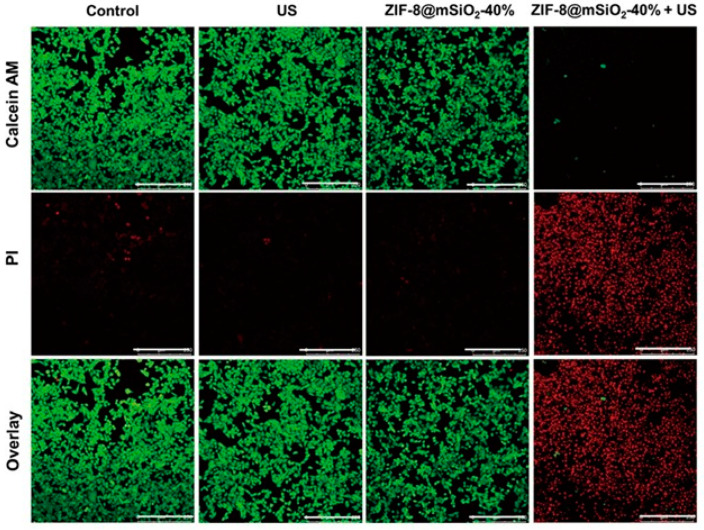
4T1 Cell viability at various concentrations of ZIF-8@mSiO_2_-40% (3.125, 6.25, 12.5, 25, 50 mg mL^−1^) with or without US treatment (1.5 Wcm@2, 1 MHz, 1 min, 50% duty cycle). Reproduced with permission from ref. [101] (copyright 2021, Wang).

Not only has the development of micro/nanotechnology created MOFs in the form of carriers, but a variety of multifunctional MOF-based nanoplatforms have also been designed to suit different situations in tumor therapy. Compared with direct drug delivery, a nano-integrated platform can improve the stability of MOFs and prolong blood circulation via ligand bonding or covalent bonding and has a specific targeting ability to enhance the efficiency of tumor therapy. In 2021, Zhang’s team loaded an alkyl radical generator (AIPH) onto a Zr-MOF backbone to construct a Zr-MOF@AIPH nanoplatform that kills tumor cells under normoxic and hypoxic conditions [100]. Meanwhile, the N_2_ gas generated from the decomposition of AIPH lowers the cavitation threshold and improves the effect of acoustic cavitation, thus enhancing the penetration of NPs at tumor sites. In vitro and in vivo studies demonstrated the superior anti-tumor efficacy, critical biocompatibility, and good imaging capability of Zr-MOF@AIPH [100]. Due to their multi-functionality, the yolk–shell nanostructures based on MOFs have received much interest. However, their formation mechanism is not yet precisely known, and there are still limitations on the regulation of yolk–shell nanostructure size and morphology. To address this issue, in 2021, Weng’s team demonstrated for the first time a solvent-dependent adsorption-driven mechanism for the synthesis of yolk–shell MOFs nanomaterials with size- and morphology-tunable mesoporous SiO_2_ shells (ZIF-8@mSiO_2_) (Figure 10) [101]. The core size and morphology of the composite can be adjusted by controlling the CH_3_OH:H_2_O ratio, mainly due to the good adsorption ability of ZIF-8 on methanol. It has been shown that nanostructures with large mesopore diameters or cavity sizes have a high cavitation effect. The yolk–shell nanostructures of ZIF-8@mSiO_2_ materials have suitable mesoporous structures and large cavity sizes and thus can undergo cavitation effects under the irradiation via US, thus exhibiting highly efficient acoustic dynamic properties for the generation of ROS, and are considered to be excellent sonosensitizers. Using electron spin resonance (ESR) analysis and the fluorescence probe method, researchers investigated the SDT properties of ZIF-8@mSiO_2_ nanomaterials, probing their ability to generate ^1^O_2_ and ·OH under the action of US. The results are shown in Figure 11. Under ultrasound, ZIF-8@mSiO_2_ nanomaterials could generate large amounts of ^1^O_2_ and ·OH. Subsequently, they also investigated the in vitro SDT performance of ZIF-8@mSiO_2_ nanomaterials. After incubation of mouse breast cancer cells (4T1 cells) under different conditions, the cells were stained using calcein acetoxymethyl ester (calcein-AM) and propidium iodide (PI) staining, followed by red fluorescence and green fluorescence, with red fluorescence showing dead cells and green fluorescence showing live cells. As shown in Figure 12, the cells treated with ZIF-8@mSiO_2_ + US showed more red fluorescence compared with other experimental controls, which indicated that the ZIF-8@mSiO_2_ nanocomposite was able to kill cancer cells in the presence of US and showed excellent SDT performance.

**Figure 13:**
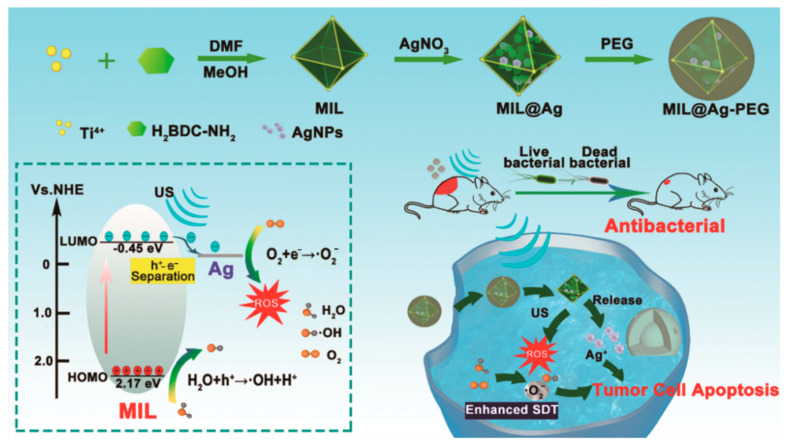
Fabrication of MIL@Ag-PEG and the MIL@Ag-PEG-Mediated Augmented SDT Mechanism for Cancer Therapy and Fast Wound Healing. Reproduced with permission from ref. [102] (copyright 2022, Meng).

Although MOFs have essential applications in SDT, many materials tend to be inefficient in their ligand–metal charge transfer, strongly affecting the SDT therapeutic efficacy. To solve this problem, in 2023, Meng’s team synthesized MIL@Ag materials via in situ modification of metallic silver NPs on the surface of Ti-MOFs [102]. In order to enhance biocompatibility and structural stability, they used PEG material to wrap it, thus obtaining MIL@Ag-PEG composite (Figure 13). The advantage of the composite is that by doping Ag NPs into Ti-MOFs, a Schottky junction is formed between Ag and Ti-MOFs, thus forming a Schottky barrier and inhibiting the recombination of carriers [102]. Notably, the charge of Ti metal in Ti-MOFs (MIL) can be transferred to Ag NPs via metal–metal transfer under ultrasound, thus alleviating the problem of inefficient ligand–metal charge transfer. The doping of Ag NPs makes the band gap of MIL narrower, and the relatively narrow band gap facilitates easier excitation and separation of electron–hole pairs, effectively eases the recombination of electron–hole pairs, generates efficient charge reactions, and improves the ability to generate ROS. To verify this theory, in subsequent experiments, MIL@Ag-PEG in vitro ROS generation efficiency was investigated using different trapping agents and fluorescent probes, and the results showed that MIL@Ag-PEG composites can significantly increase ROS generation efficiency and have the potential to improve SDT therapeutic efficacy. In an in vitro anti-tumor assay, the researchers first investigated whether the MIL@Ag-PEG composite could be effectively endocytosed by cancer cells by labeling MIL@Ag-PEG with Cy3 fluorescent dye and then incubating with MCF-7 cells, followed by instrumental observation of the fluorescence. The results showed that the intensity of intracellular Cy3 fluorescence was gradually increased with incubation time, confirming that MIL@Ag-PEG composite material can be effectively endocytosed by cancer cells. Subsequently, they investigated the in vitro anticancer effects of MIL@Ag-PEG materials via the 3-(4,5-dimethyl-2-thiazolyl)-2,5-diphenyl-2-H-tetrazolium bromide (MTT) method. The findings demonstrated that MIL@Ag-PEG has significant anti-cancer properties and could considerably harm MCF-7 and A549 cells in the presence of ultrasound. The researchers covered A549 cells with lean meat to imitate the tumor environment at various depths to assess the potential of MIL@Ag-PEG composites in deep tumor therapy. The researchers then used ultrasound to irradiate MIL@Ag-PEG-incubated cancer cells and showed that SDT caused damage to cancer cells even at a barrier thickness of 2 cm, demonstrating the good penetration ability of SDT, which is also more suitable for deep cancer treatment than other treatment methods. The results obtained from the in vivo experiments in mice demonstrate the high biocompatibility of MIL@Ag-PEG composites and their ability to be activated by highly penetrating US to improve the therapeutic effect of SDT. Finally, the team evaluated the antimicrobial efficacy of MIL@Ag-PEG composites for SDT treatment. MIL@Ag-PEG has high biocompatibility and biosafety in antibacterial treatment, and is not easy to cause inflammation. In summary, MIL@Ag-PEG composite material prepared by Meng’s team has good efficiency in electron transfer and O_2_ adsorption capacity, which gives novel ideas for improving the efficacy of SDT and has a good application prospect.

**Figure 14:**
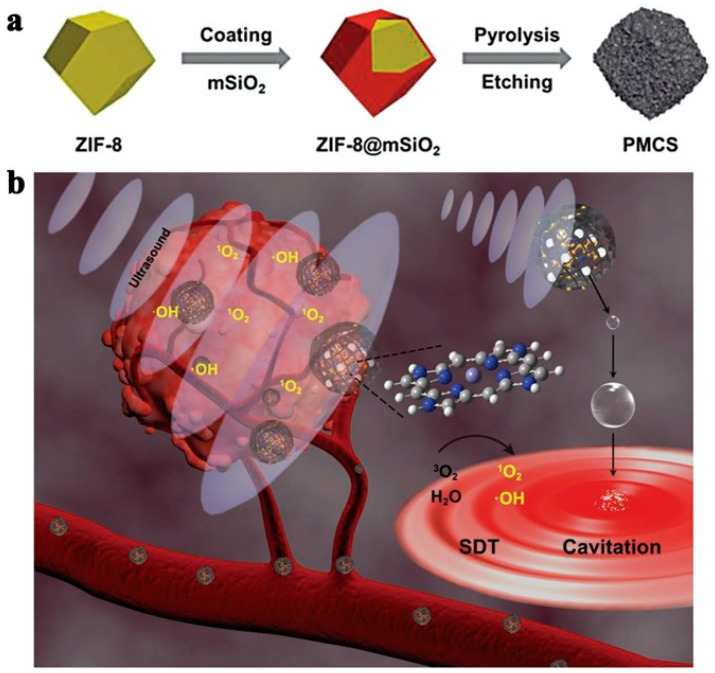
(**a**) PMCS synthesis and characterizations, (**b**) PMCS sonosensitization process for cancer therapy. Reproduced with permission from ref. [103] (copyright 2018, Pan).

Pan’s team synthesized MOF-derived mesoporous carbon nanostructures (PMCS) containing porphyrin-like metal centers using the metal–organic skeleton ZIF-8 as the main body via high-temperature calcination and applied them in SDT (Figure 14) [103]. The researchers determined the enhancement effect of PMCS on SDT effect by detecting the generation of ^1^O_2_ and ·OH using electron spin resonance (ESR). To see the effect of ^1^O_2_ production, they used porphyrin Zn and mesoporous carbon nanospheres (MCN) as the control group, followed by mixing porphyrin Zn, MCN, and PMCS with 2,2,6,6-tetramethylpiperidine, respectively, followed by irradiation of each group of samples using US. The results showed a 3.2 times increased peak intensity of PMCS compared to porphyrin Zn and 116.5% compared to MCN. This indicates that the PMCS material can effectively enhance the generation ^1^O_2_, thus strengthening the SDT effect. In addition, they also investigated the cavitation effect of PMCS and compared the cavitation clusters in water and PMCS solution under the same super conditions. The results show that the number of cavitation bubbles in PMCS is more extensive than in water, and the volume of the expanded bubbles is generally more significant than the volume of bubbles in the water. These results suggest that PMCS enhances the cavitation effect of ultrasound due to PMCS’s high effective surface area and a porous structure that allows it to bind to US and thus exhibit a stronger cavitation effect. In cellular experiments, they treated and observed the cells after co-incubating PMCS with 4T1 for 24 h. The results showed that PMCS could effectively endocytose into cancer cells. To investigate the toxicity of PMCS on cells, the 4T1 cells’ viability was determined after incubation with various PMCS concentrations for 24 h using a standard methyl thiazolyl tetrazolium assay. Even at high concentrations of 100 µg/mL, PMCS did not produce significant cytotoxicity. However, under US irradiation, 4T1 cells’ activity decreased with increased PMCS concentration. These results indicate that PMCS has good biocompatibility and high acoustic toxicity, which provides an important reference for developing sonosensitizers agents and efficiently treating cancer.

**Figure 15:**
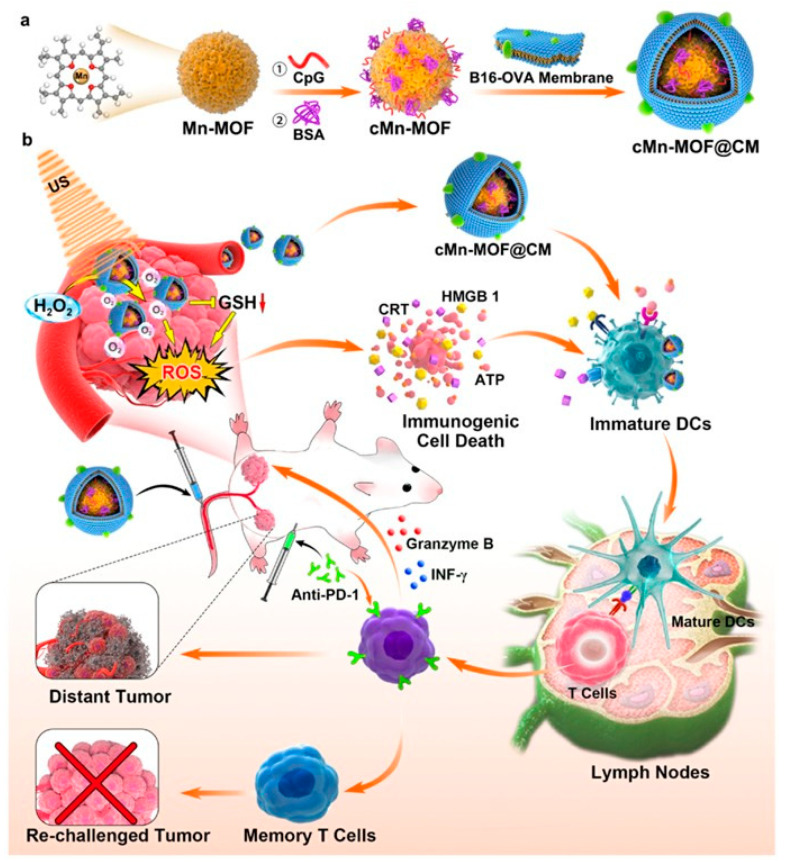
(**a**) Schematic illustration of the preparation of cMn-MOF@CM. (**b**) Schematic illustration of cMn-MOF@CM-triggered SDT and nanovaccine to improve anti-PD-1 efficacy. cMn-MOF@CM with prolonged blood circulation and enhanced tumor targeting efficiently relieves tumor hypoxia and decreases intracellular GSH, generating strong SDT effects and ICD. The tumor-associated antigens both in situ derived from SDT and OVA exhibit vaccine-like functions together with the immune adjuvant CpG, promoting DC maturation and T cell activation. Combined with anti-PD-1 antibody, cMn-MOF@CM with US irradiation generates stronger long-term memory immunity. Reproduced with permission from ref. [104] (copyright 2021, Zhan).

In 2021, Zhan’s team combined Mn-MOF with toll-like receptor 9 (TLR9) agonist CpG oligodeoxynucleotides via electrostatic adsorption [104]. Then it was coated with cancer cell membrane (CM) from mouse melanoma B16 cells overexpressing ovalbumin (OVA) antigen to construct a bionic nanoplatform of cMn-MOF@CM (Figure 15) [104]. The cMn-MOF@CM nano mimetic platform can produce a strong SDT effect and effectively alleviate hypoxia in the tumor microenvironment. In addition, it can also effectively enhance the targeting and immunogenicity to tumors. More importantly, the cMn-MOF@CM-triggered SDT/anti-pd-1 antibody combination implies a more powerful systemic immune response and long-term immune memory function for preventing the growth and recurrence of tumors. In animal experiments, cMn-MOF@CM anticancer activity, as an SDT inducer and nanovaccine, was found by injecting subcutaneous B16-OVA melanoma mice intravenously with PBS, Mn-MOF, cMn-MOF, Mn-MOF@CM, or cMn-MOF@CM, respectively. In the absence of ultrasound, only the cMn-MOF @CM group exhibited an inhibitory tumor growth, while the other groups did not significantly affect tumor growth. This confirmed the function of cMn-MOF@CM as a nano-vaccine. In the presence of ultrasound, Mn-MOF was able to significantly inhibit tumor growth, while the cMn-MOF@CM group showed the best tumor inhibition. Notably, when mice treated with ultrasound injected with cMn-MOF@CM were removed from residual tumors and attacked again with B16-OVA cells, no visible secondary tumor growth was observed for 23 days. This demonstrated that cMn-MOF@CM exhibited boosted anticancer activity of nanovaccines and SDT inducers by generating immune memory. The primary mechanism is that cMn-MOF@CM significantly inhibited dendritic cell (DC) maturation within the tumor with or without US irradiation. Under the effect of US, CD8^+^CD44^+^CD62L^−^ effector memory T cells were critically enhanced in the spleen and tumor-draining lymph nodes of the cMn-MOF@CM group, which is the main reason why cMn-MOF@CM can effectively induce immune memory. The above research results suggest that the cMn-MOF@CM nano mimetic system, a potential drug for combining immune checkpoint blockade therapy (ICB) and SDT, is significant.

**Figure 16:**
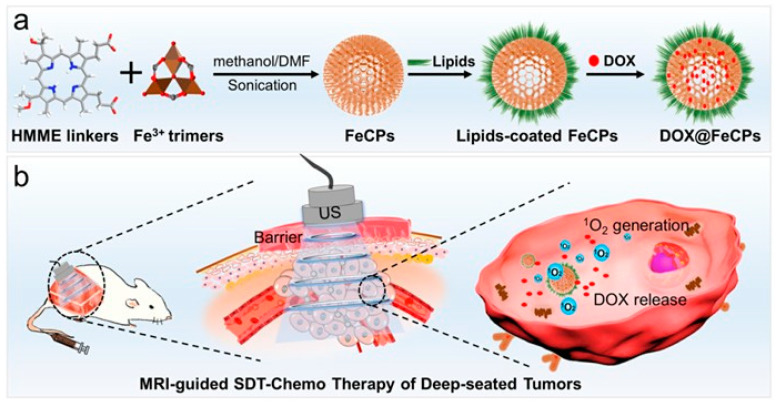
(**a**) The DOX@FeCPs synthesis. (**b**) DOX@FeCPs intravenously administration for MRI-guided SDT-chemo therapy on deep-seated tumor model. Reproduced with permission from ref. [106] (copyright 2020, Xu).

Xu’s team also used Mn-MOF to construct a sonosensitizer that continuously catalyzes the in situ production of O_2_ from tumor-overexpressed H_2_O_2_ to alleviate tumor hypoxia [105]. The results showed strong anticancer and antimetastatic activities in H22 and 4T1 tumor-bearing mice after a single Mn-MOF administration following a single US irradiation. Mn-MOF effectively reshapes the tumor immune microenvironment under US irradiation by increasing activated CD8^+^ T cell numbers and mature DCs and reducing the number of MDSCs in tumor tissue, with strong potential as an advanced hypoxic cancer therapy system [105]. SDT using semiconductor or organic sonosensitizer is gaining increasing attention as a non-invasive treatment for deep-seated tumors. However, its practical application is still limited due to unsatisfactory treatment effects. As a new second-generation porphyrin-related sonosensitizer, hematoporphyrin monomethyl ether (HMME) hematoporphyrin derivatives (HpD), consists of two monomeric porphyrins with two carboxyl groups and the carboxyl group of HMME is feasible as a bridging linker for coordinating metal ions to build new metal–organic nanostructures. To address the problems of existing sonosensitizer in SDT, Xu’s team reported a metal–organic nanosensitizer that assembled the drug HMME with Fe^3+^ ions through covalent coordination, resulting in nanoscale Fe-HMME particles (FeCP). FeCP nanoparticles are not only good new sonosensitizers, capable of producing ^1^O_2_ under the action of US, but also excellent drug carriers, forming a multifunctional platform of DOX@FeCPs after carrying the anti-cancer drug DOX (Figure 16) [106]. The excellent effective surface area and porosity give FeCPs a high DOX loading capacity (31.3%) and the ability to slow-release DOX intracellularly, critically inhibiting the growth of deep tumor models and enabling effective SDT chemotherapy combination therapy. Iron-based nanomaterials, such as Fe_3_O_4_ and Fe-MOFs, have been broadly used as MRI contrast agents for T1/T2-weighted imaging.

In the same way, researchers have experimentally confirmed that FeCPs are also an MRI contrast agent with contrast imaging properties, thus serving to monitor the therapeutic effects of drugs in vivo in real-time. In an experiment to investigate the effect of DOX@FeCPs nanotherapeutic platform on SDT of deep tumor tissues, DOX@FeCPs was injected intravenously into mice with CT26 tumors covered by a 2-cm barrier. Then the effect of SDT was studied in vivo and showed that DOX@FeCPs had a sound impact on SDT and chemotherapy in deep tumor models. Thus, DOX@FeCPs nanotherapeutic platform can effectively mediate SDT and has good therapeutic properties against deep tumors, which provides essential ideas for the design of other metal–organic nanoplatforms for SDT.

Hoang’s team developed a nanoscale zirconium-based porphyrin MOF (PCN-222) as a safe and effective nanosensitizer [107]. To improve the stability of the material as well as its biocompatibility, the researchers used polyethylene glycol (PEG) to couple with PCN-222, and to improve the therapeutic ability of this nanotherapeutic system for tumors, they used polyethylene glycol (PEG)-encapsulated PCN-222 (PEG-PCN) to carry the pro-oxidant drug piperidone (PL), to enable tumor-specific chemo-photodynamic combination therapy. It was shown that nanoscale PL-PEG-PCN was effectively internalized by breast carcinoma cells and that the effect of US significantly increased ROS production. Comparison of the cytotoxicity of PEG-PCN and PCN-222 on MCF-7 revealed that the cell viability of PEG-PCN was higher than that of PCN-222, and the cell survival rate of PEG-PCN-treated cells was higher than 90% when the concentration was 20 µg/mL, which indicated that the PEG wrapping significantly enhanced the biocompatibility of the composite. To confirm the apoptosis induced by SDT via PCN-222-based NPs, MCF-7 cells were incubated with PEG-PCN and PL-PEG-PCN, respectively, and subsequently assessed via flow cytometry and membrane-linked protein V/PI staining for apoptosis. The results showed a high apoptosis rate in cells receiving PL-PEG-PCN under irradiation with US, which could be attributed to the oxidative stress effect induced by PEG-PCN under irradiation with US and the caspase-dependent apoptosis of human breast carcinoma cells induced by PL via inhibition of the PI3K/Akt/mTOR signaling pathway. The successful construction of PL-PEG-PCN illustrates that nanoporphyrin MOF, in combination with pro-oxidant drugs, is a safe and effective nanosensitizer for use in efficient SDT. Similarly, Ren’s team constructed a DOX@PCN-224/Pt nanotherapeutic platform by in situ reduction of Pt nanoclusters (1.5 nm) in porous structures using porphyrin-based MOFs (PCN-224) as the main body, followed by loading of the anticancer drug DOX (loading efficiency of 26.3%) [108]. To address the problems of tumor microenvironment hypoxia and the relatively inefficient ability to exist acoustic sensitizers to generate ^1^O_2_, the presence of Pt nanozymes could effectively catalyze the conversion of H_2_O_2_ to O_2_, which could not only promote cytotoxic ^1^O_2_ production by PCN-224 under US irradiation but also significantly downregulate the expression of HIF-1α to improve the tumors’ sensitivity to chemotherapy, resulting in efficient tumor inhibition. When DOX@PCN-224/Pt NPs were injected into negative tumor mice, the material showed efficient ^1^O_2_ production ability as well as DOX chemotherapeutic efficacy. This nanozyme-enhanced tumor treatment strategy offers new application prospects for broadening the application of nanozyme.

### 2.3. Combined Treatment of SDT Based on MOF-Sonosensitizer

**Figure 17:**
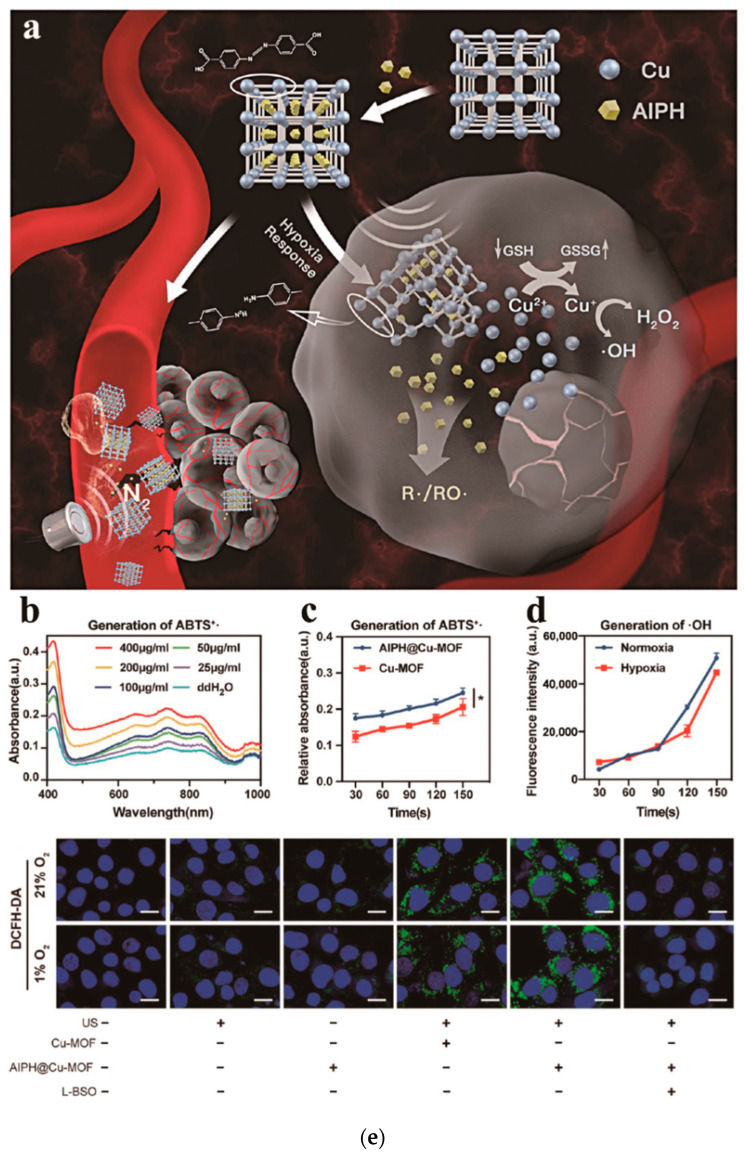
Generation of free radicals via US irradiation. (**a**) UV–vis spectra of ABTS^+•^ produced after combining ABTS with various concentrations of AIPH@Cu-MOF. (**b**) Absorbance of ABTS^+•^ at 736 nm in US-irradiated AIPH@Cu-MOF or Cu-MOF solutions. (**c**) Quantification of •OH produced by AIPH@Cu-MOF under normoxic or hypoxic conditions, as detected with APF. * *p* < 0.05. (**d**) Levels of intracellular free radicals in Panc02 cells determined by the DCFH-DA probe. (**e**) The cells were treated with AIPH@Cu-MOF (25 μg/mL), Cu-MOF (25 μg/mL), US (1.0 MHz, 0.5 W/cm^2^, 2 min, 50% duty cycle), or L-BSO (50 μg /mL). Scale bar: 20 μm. Reproduced with permission from ref. [109] (copyright 2021, Sun).

SDT’s therapeutic efficacy is often influenced by the oxygen content of the body’s environment, where the hypoxic microenvironment significantly limits therapeutic efficacy. Therefore, the development of oxygen-independent free radical generators and synergistic effects in combination with other therapeutic approaches can potentially enhance the antitumor efficacy of SDT. AIPH@Cu-MOF nanoparticles were synthesized by doping AIPH into Cu-MOF [109]. The nanocomposite can be effectively combined with SDT and CDT for synergistic cancer treatment. The main mechanism is that the Cu-MOF structure collapses when the AIPH@Cu-MOF nanocomposite reaches the tumor microenvironment. The loaded ALPH is released, and Cu is converted to Cu^2+^, which can react with glutathione (GSH) in the tumor microenvironment to form Cu^+^, which in turn can react with endogenous hydrogen peroxide to yield Cu^+^ which can, in turn, respond with endogenous hydrogen peroxide to yield ·OH, which is potent against cancer cells. Under the action of ultrasound, the released AIPH was excited to yield a large number of nitrogen bubbles and alkyl radicals, which induced a large amount of cancer cell DNA damage and cancer cell death, and the N_2_ bubbles produced by AIPH could further improve the penetration ability of AIPH@Cu-MOF nanocomposites. Thus, the main advantage of AIPH@Cu-MOF nanocomposites lies in its ability to achieve combined CDT and SDT therapy in a hypoxic tumor microenvironment and good target penetration capabilities. In the experimental part, the researchers evaluated the ability of AIPH@Cu-MOF nanocomposites to generate free radicals under the action of ultrasound by observing the amount of ABTS reacting with free radicals to produce ABTS^+·^. Based on Figure 17a,b, the production of ABTS^+·^ was increased with the increase in AIPH@Cu-MOF concentration, and the ABTS^+·^ absorbance of the AIPH@Cu-MOF treated group was significantly higher than that of the Cu-MOF treated group, which indicates that AIPH was able to effectively decompose the free radicals generated under the action of ultrasound. The results are shown in Figure 17c; the amount of ·OH produced by AIPH@Cu-MOF nanocomposites under a normal oxygen concentration environment and low oxygen environment shows almost no difference, which indicates that AIPH@Cu-MOF nanocomposites can produce ·OH under low-oxygen environment conditions, thus causing tumor death. The results of the intracellular free radical fluorescent labeling experiment are shown in Figure 17d. This resulted in the ability of the AIPH@Cu-MOF nanocomposite to generate free radicals intracellularly under ultrasound. Subsequently, the researchers also evaluated the anti-tumor performance of AIPH@Cu-MOF nanocomposites in synergy with CDT and SDT and the effect of this combination therapy on the intra-tumor immune response. From all the experimental data, it can be gleaned that AIPH@Cu-MOF nanocomposites have excellent penetration ability and can effectively bind CDT and SDT in the hypoxic tumor microenvironment with synergistic anti-tumor action, which is of great clinical significance.

**Figure 18:**
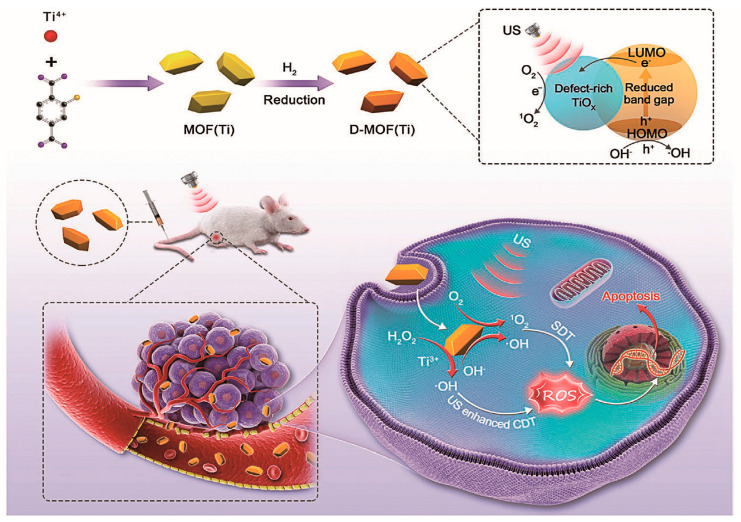
Schematic for D-MOF(Ti) synthesis and antitumor therapy. Reproduced with permission from ref. [110] (copyright 2021, Liang).

**Figure 19:**
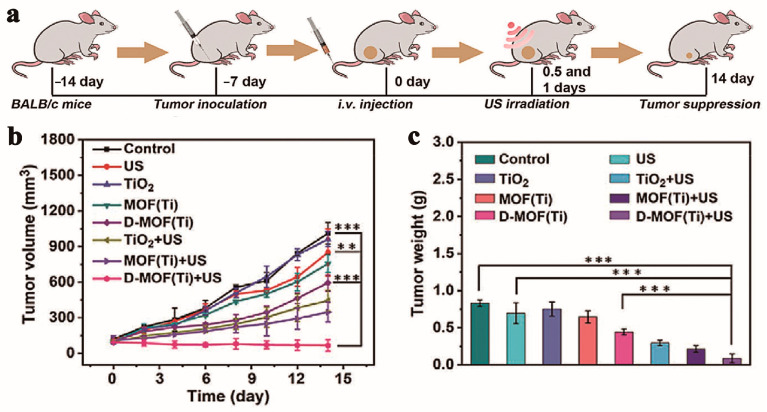
(**a**) Schematic for illustrating tumor treatment. (**b**) Curves of body weight and (**c**) tumor volume of mice treated with various treatments (mean ± S.D., n = 5, one-way analysis of variance (ANOVA) with Dunnett’s post-hoc test, ** *p* < 0.01, and *** *p* < 0.001). Reproduced with permission from ref. [110] (copyright 2021, Liang).

In 2021, Liang’s team first prepared common Ti-based MOFs (NH_2_-MIL-125) vai a simple synthesis method and then reduced them using H_2_, resulting in rich defects on their surfaces, thus obtaining D-MOF(Ti) [110]. The D-MOF(Ti) synthesis and antitumor therapy are shown in Figure 18. Compared with NH_2_-MIL-125 without H_2_ treatment, D-MOF(Ti) has a narrower band gap structure, which helps to generate more ROS under the action of US, while effectively improving the electron–hole separation triggered by ultrasound. In addition, because titanium is an excellent biocompatible metal, the D-MOF(Ti) material possesses low toxicity to humans. It has high biocompatibility, as demonstrated experimentally by researchers who injected D-MOF(Ti) NPs (dose: 15 mg kg^−1)^ intravenously into mice and continuously measured routine blood indicators at the liver for 30 days. The toxicity of D-MOF(Ti) nanoparticles was evaluated via measuring blood parameters and liver parameters for 30 days. Due to the presence of Ti^3+^, D-MOF(Ti) can undergo a Fenton reaction in the tumor microenvironment, resulting in CDT. In an in vivo anti-tumor experiment, eight experimental groups were designated to evaluate the effect of D-MOFs(Ti) combined with CDT and SDT in treating tumors. The results are shown in Figure 19. In the absence of US, D-MOFs(Ti) still had an inhibitory tumor growth effect, mainly because of the role played by D-MOF(Ti)-mediated CDT. Compared with other experimental groups, D-MOF(Ti) + US was the most effective in inhibiting tumor growth. With its simple structure, excellent biocompatibility, and synergistic cancer treatment, this nanocomposite is very promising for clinical applications.

**Figure 20:**
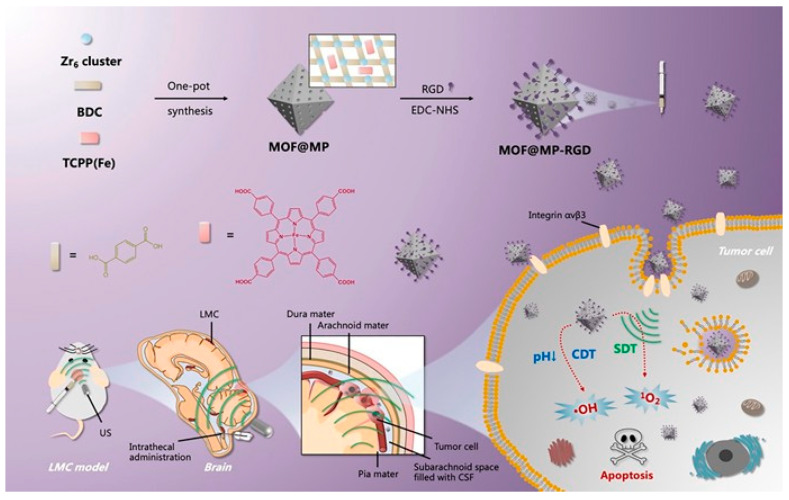
MOF@MP-RGD nanosystem preparation and the mechanism of combining SDT/CDT effect for treating LMC by intrathecal drug delivery. Reproduced with permission from ref. [111] (copyright 2022, Niu).

**Figure 21:**
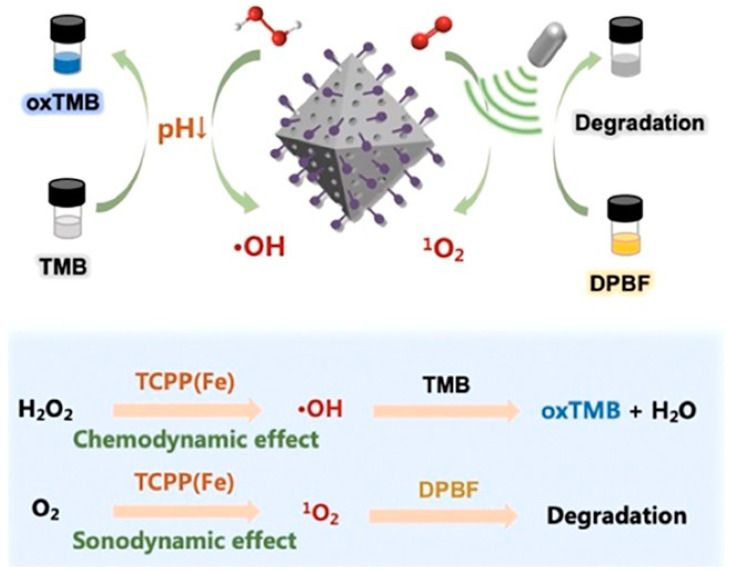
Mechanistic illustration of ROS generation via MOF@MP-RGD nanosystems based on Fenton-like reaction and US irradiation. Reproduced with permission from ref. [111] (copyright 2022, Niu).

**Figure 22:**
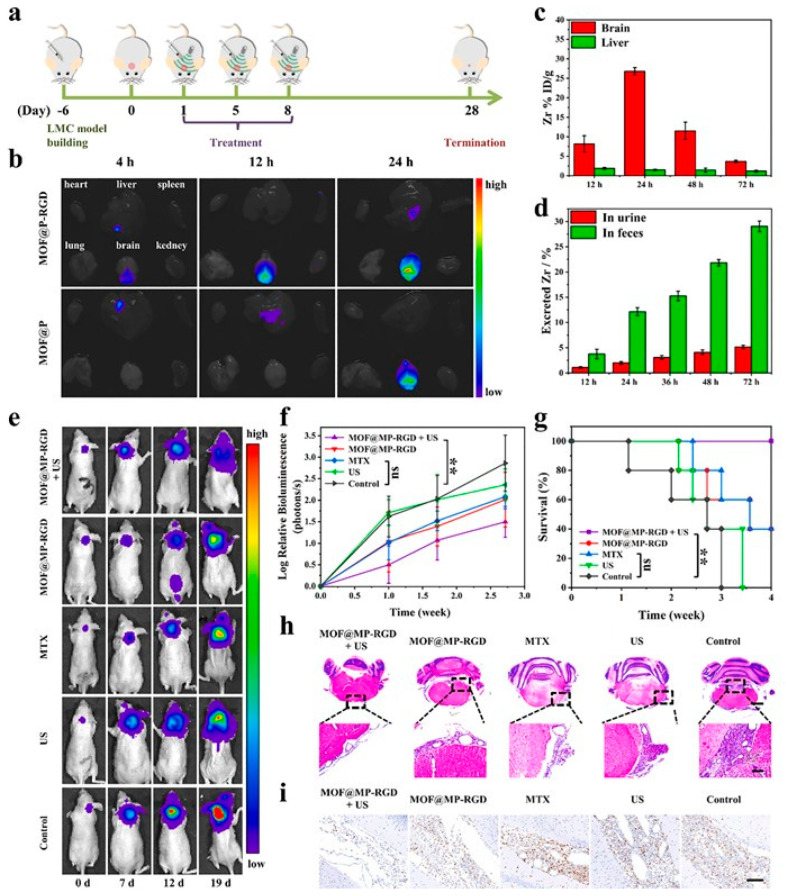
In vivo biodistribution and antitumor properties of MOF@MP-RGD nanosystems. (**a**) In vivo therapeutic protocol of MOF@MP-RGD nanosystems with US irradiation on the orthotopic LMC mice. (**b**) Ex vivo fluorescence imaging of heart, liver, spleen, lung, brain, and kidney of the LMC mice at different time points after being intrathecally injected with MOF@P and MOF@P-RGD nanosystems (dose: 50 µg). (**c**) Biodistribution of Zr (% injected dose (ID) of Zr per gram of tissues) in the brain and liver of LMC mice at different time points after being intrathecally injected with MOF@MP-RGD nanosystems (n = 3). (**d**) Accumulated Zr (in urine and feces) excreted out of the LMC mice after intrathecal administration of MOF@MP-RGD nanosystems (dose: 50 µg) for different durations (n = 3). (**e**) Representative images of time-dependent luciferin fluorescence imaging for the LMC mice after different treatments. (**f**) Tumor growth curves reflected by the change of tumor luciferin signal intensities in the LMC mice of different treated groups (n = 5). At day 19, the significantly lower luciferin signals were detected in the MOF@MP-RGD + US group than in the control group (*p* = 0.00782 (**), Student’s *t* test). Besides, the luciferin signals in the MTX group were not significantly lower than in the control group (*p* = 0.07952 (ns), Student’s *t* test). (**g**) Kaplan-Meier survival curves of the LMC mice after different treatments. All animals in the MOF@MP-RGD group survived >4 weeks after treatment, significantly better than the control group (*p* = 0.003 (**), Log-rank test), whereas no significant difference was found between the MTX group and the control group (*p* = 0.055 (ns), Log-rank test). (**h**) H&E (cerebellar level, scale bar (above) = 1000 µm, scale bar (below) = 100 µm) and (**i**) Ki-67 (scale bar = 100 µm) staining of the tumor tissues from different groups. Reproduced with permission from ref. [111] (copyright 2022, Niu).

In 2022, Niu’s team synthesized MOF@MP-RGD nanoparticles with synergistic SDT/CDT capability via the solvothermal method for intrathecal injection targeting leptomeningeal carcinomatosis (LMC) (Figure 20) [111]. The introduction of arginine-glycine-aspartic acid (RGD) enhances the targeting of the nanomedicine, and the intrathecal administration can effectively overcome the barrier associated with the off-brain, which facilitates the MOF@MP-RGD nanomedicine to reach the deeper source of the disease and produce more efficient therapeutic effects. The MOF@MP-RGD nanosystem is rich in Fe (III) atoms, which can act as a catalytic center to convert hydrogen peroxide to the highly toxic ·OH via a Fenton-like reaction (Figure 21). The Michaelis–Menten curves were plotted and the maximum velocity (V_max_) and Michaelis–Menten constant (K_M_) were determined as 6.45 × 10^−8^ M·S^−1^ and 1.33 × 10^−4^ M, respectively, which indicated the excellent CDT performance of MOF@MP-RGD nanomaterials via experimental test. The SDT performance of MOF@MP-RGD nanoparticles was evaluated using a DPBF probe. The characteristic absorbance of DPBF did not change significantly when only MOF@MP-RGD nanoparticles were present, while the characteristic absorbance of DPBF was critically decreased within 8 min in the presence of US + MOF@MP-RGD nanoparticles simultaneously, which reflected the SDT performance of MOF@MP-RG—in vitro experiments demonstrated that the MOF@MP-RGD nanosystem has high biocompatibility and obtained desirable therapeutic effects due to the production of large amounts of ROS via combined chemical kinetic/acoustic kinetic effects, which induced amplification of oxidative stress in tumor cells. Through the establishment of an in situ LMC tumor model (Figure 22), it was confirmed that the MOF@MP-RGD nanosystem accumulated significantly in LMC tumor tissues after intrathecal administration into the cerebrospinal fluid and was able to be excreted via metabolic processes.

**Figure 23:**
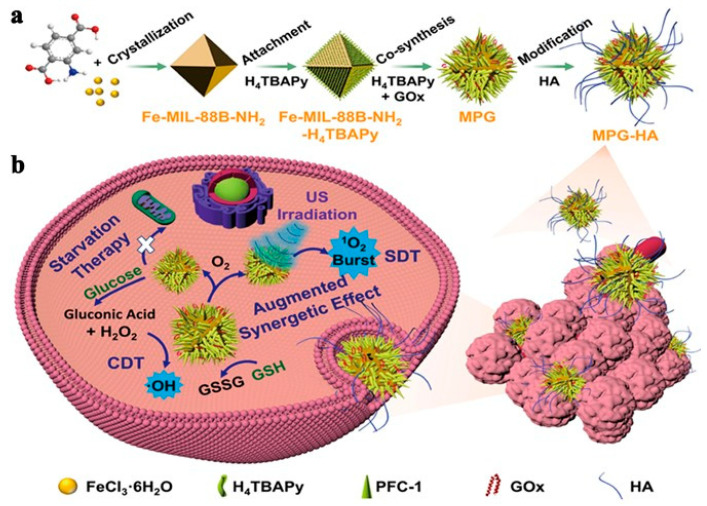
(**a**) MPG-HA NPs synthesis and (**b**) Anticancer Mechanism of MPG-HA-Based Synergetic CDT/SDT/Starvation Therapy. Reproduced with permission from ref. [112] (copyright 2021, Hu).

**Figure 24:**
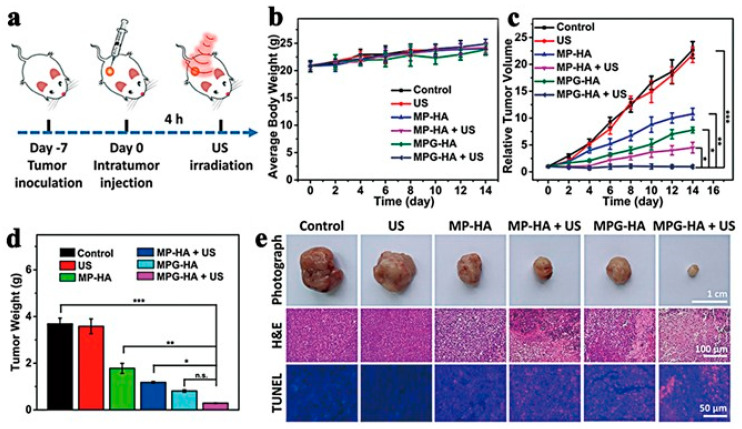
(**a**) The in vivo antitumor effect of MPG-based boosted therapy. (**b**) Weight of body, (**c**) volume of tumor, and (**d**) change in tumor weight curves of Balb/C mice after 14 days of treatment with different options. (**e**) Representative photographs, H&E staining, and TUNEL staining images of tumors for all groups. MP-HA or MPG-HA dose: 100 μL (800 μg mL^−1^) for each mouse. Data: mean ± SD (n = 3); * *p* < 0.5; ** *p* < 0.01; *** *p* < 0.001; not significant = n.s. Reproduced with permission from ref. [112] (copyright 2021, Hu).

Tumor cells often need to absorb external nutrients to grow, and the starvation therapy emerging in recent years can “starve” tumor cells by blocking their nutrient supply. The therapeutic strategy of starvation therapy mainly consists of interfering with the mechanism of tumor angiogenesis by targeting the inhibition of pro-angiogenic factors and their receptors and integrins to combat tumor angiogenesis; by embolizing and squeezing the blood vessels, the oxygen-supplying function of tumor vessels was blocked. The metabolic process of tumor cells was inhibited by inhibiting serine/glycine/carbon metabolism, glycolysis and amino acid metabolism of mitochondria. In recent years, the boosted treatment of tumors has been achieved by combining starvation therapy with SDT. In 2021, Hu’s team constructed Fe-MIL-88B-NH_2_@PFC-1-GO_x_ (MPG) NPs based on Fe-MIL-88B-NH_2_, PFC-1 (HOF-101) and glucose oxidase (GO_x_) [112]. To improve the structural stability of MPG nanoparticles, the researchers used hyaluronic acid (HA) to coat the MPG material (Figure 23). MPG NPs can effectively combine CDT/SDT/starvation therapies to inhibit tumor cell growth synergistically. The main principle is that MPG nanoparticles exhibit catalase (CAT), glutathione (GSH) peroxidase, and Fenton activities because of the presence of Fe^3+^/Fe^2+^ redox pair, so MPG can generate ·OH for CDT and deplete glutathione (GSH) to reduce the antioxidant capacity of cancer cells. In addition, MPG can react with H_2_O_2_ to produce O_2_, thus alleviating hypoxia in the microenvironment of tumor and enhancing the effect of GO_x_ catalyzing the oxidation of glucose to produce hydrogen peroxide and gluconic acid, thus cutting off the energy source of tumor cells and achieving starvation treatment. The regenerated H_2_O_2_ can improve the Fenton reaction, resulting in GO_x_-catalyzed enhanced CDT. Due to its large π-electron conjugation system, MPG is also an ideal sonosensitizer for efficient SDT with burst production of ^1^O_2_ under US irradiation. Researchers have explored the hemolysis rate and the effect of MPG-HA nanoparticles on red blood cell morphology in a physiological environment, it was obtained that the hemolysis rate of MPG-HA was lower than 9% (3.13–800 μg mL^−1^), and the morphology of erythrocytes was not significantly changed, which further indicated that MPG-HA nanoparticles had good biocompatibility and biosafety. Researchers used a 4T1 xenograft tumor model in mouse studies to examine the impact of MPG-HA on tumor inhibition in vivo (Figure 24). The MP-HA group and MPG-HA showed significant tumor growth inhibition, mainly because of the former’s ability to produce a significant CDT effect and the latter’s ability to have CDT/starvation treatment effect. Under the action of ultrasound, MPG-HA is activated to obtain the effects of SDT, which further improves tumor inhibition ability. This is because when MPG-HA is combined with US, SDT produces ^1^O_2_, thus producing the synergistic effect of CDT, SDT and starvation therapy. Subsequently, MPG-HA degradability was studied by in vitro and in vivo experiments on Fe levels in the major organs, tumors, and feces. Based on the results, the highest Fe concentration in the major organs was found 12 h after MPG-HA injection, followed by a gradual decrease. At the same time, Fe was detected in feces, confirming that MPG-HA can be effectively degraded and eliminated. Blood biochemical and hematological analyses were performed on mice treated with PBS and MPG-HA + US. The results showed that all the measured indexes were within the reference values’ range, which indicated that MPG nanoparticles caused little damage to normal tissues and had good safety in vivo application. In conclusion, the construction of MPG-HA suggests new ideas for applying MOFs combined with HOFs and enzymes as multifunctional nanoplatforms in the biomedical field and a new basis for the boosting treatment of SDT combined with starvation therapy [112].

**Figure 25:**
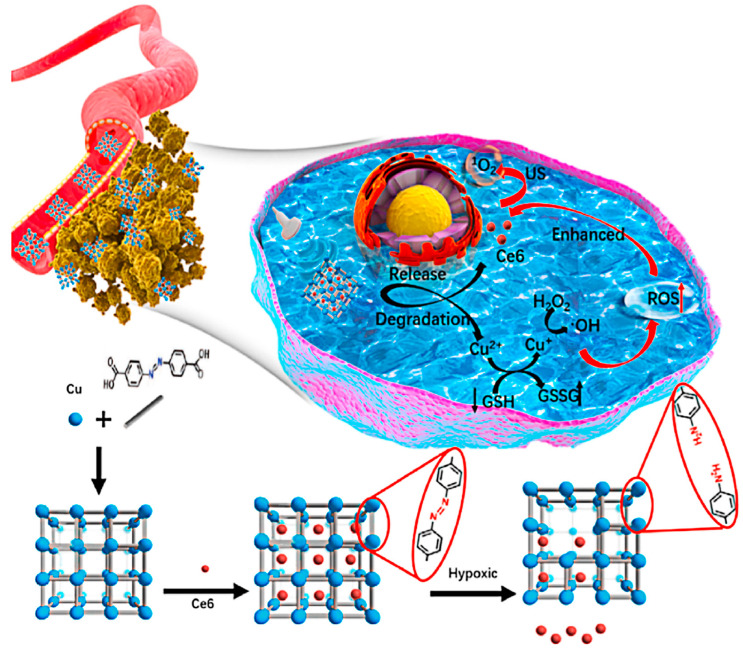
The Copper MOFs nanosystem Synthesis and Hypoxia Responsive for Enhanced Cancer Therapy. Reproduced with permission from ref. [113] (copyright 2020, Zhang).

**Figure 26:**
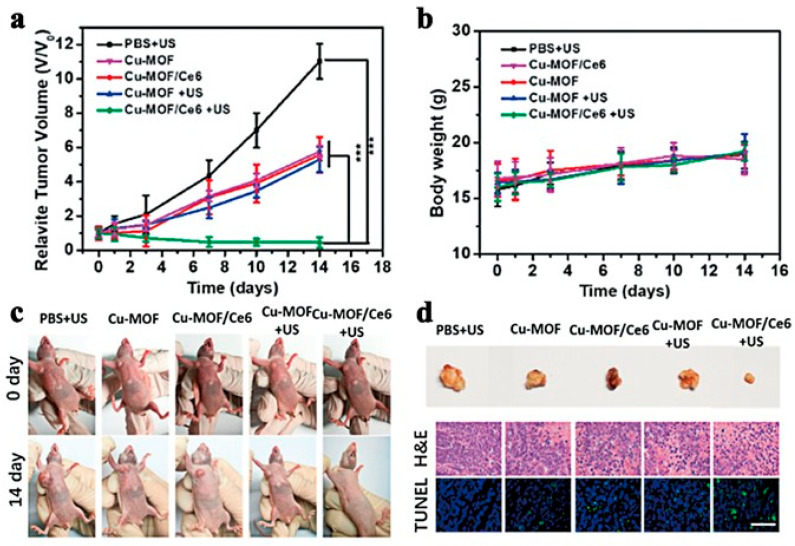
(**a**) Relative volumes of tumor and (**b**) weight of body of mice received various treatments. (**c**) Photographs of tumor-bearing mice before various treatments and after 14 days. (**d**) Photographs of tumors harvested after 14 days post-treatment. H&E staining and TUNEL staining of tumor sections from multiple groups. Scale bar: 200 μm. *** *p* < 0.001. Reproduced with permission from ref. [113] (copyright 2020, Zhang).

In 2020, Zhang’s team constructed Cu-MOF/Ce6 nanoparticles by bridging copper clusters with organic ligands loaded with the acoustic sensitizer chlorin e6 (Ce6) (Figure 25) [113]. The nanoparticles effectively achieved the effects of SDT/CDT synergistic treatment for breast cancer. Large-sized Cu-MOF/Ce6 NPs exhibit excellent stability at a normal partial pressure of oxygen and can form accumulations at tumor sites. When Cu-MOF/Ce6 nanoparticles are exposed to the hypoxic tumor microenvironment (TME), Cu-MOF/Ce6 is rapidly degraded and releases Cu^2+^ as well as the acoustic sensitizer Ce6 and penetrates deep into the tumor, where free Cu^2+^ reacts with high levels of intracellular Redox reactions between GSH lead to GSH depletion and reduction of Cu^2+^ to Cu^+^, followed by reaction of Cu^+^ with endogenous hydrogen peroxide to generate strong cytotoxic hydroxyl radicals (·OH) via Fenton-like reaction of CDT. The release of the acoustic sensitizer Ce6 in the presence of US can further mediate SDT, thus providing a synergistic treatment effect. The researchers experimentally demonstrated that Cu-MOF/Ce6 NPs could accumulate at the tumor site, has a long circulation time, has good penetration ability in a hypoxic environment and can specifically kill cancer cells. In an in vivo anticancer experiment, nude mice carrying MCF-7 cancer cells in the right forelimb were randomly divided into five groups and injected with or without US irradiation in the tail vein with PBS (10 mM, pH 7.4), Cu-MOF or Cu-MOF/Ce6 (10 mg/kg per mouse), respectively, and then observed the tumor volume changes of the tumor-bearing mice receiving different treatments over 14 days. The results are shown in Figure 26 and indicate that Cu-MOF/Ce6 plus US irradiation showed the highest ability to inhibit tumor growth and tumor growth was significantly inhibited and gradually destroyed. The construction of Cu-MOF/Ce6 nanosystem is not a new type of anoxic reactive MOF nanosystem. The system has the properties of SDT/CDT. It can be flexibly used in multi-mode therapy, which provides an important basis for designing an anoxic reactive MOF nanotherapy platform. Faced with the problem of systemic side effects of first-line antitumor drugs, Chen’s team in 2021 prepared a nanoplatform for the mitigation of hypoxia by nucleating zeolite imidazole ester backbone 90 (ZIF-90) NPs on Pt NPs and co-loading them with gemcitabine and IR780 [114]. In the presence of loaded PTs that catalyze peroxides into oxygen and address hypoxia, and the presence of gemcitabine that improves the sensitivity of tumor cells to chemotherapy, this chemo-acoustic kinetic therapy offers a promising strategy for treating tumors [114].

## 3. Conclusions and Challenges

In this article, we systematically summarized the most recent progress of SDT based on functional nanomaterials MOFs in cancer, and demonstrated how sonosensitizers can be better applied in SDT from different perspectives, providing some new ideas for subsequent cancer treatment research. A large number of experiments have confirmed that SDT, as a new non-invasive and precise tumor treatment method, takes advantage of a high concentration of sonosensitizers gathered at the tumor site and then uses specific ultrasound to activate the sonosensitizers to produce a large number of ROS to kill tumor cells after precise localization, which has the advantages of being non-invasive, targeted, highly efficient and synergistic, showing broad application prospects. However, as an essential part of SDT, most of the sonosensitizers reported today are still derived from photosensitizers, which have certain limitations, and only a few organic or inorganic materials are widely used in SDT, which dramatically limits the advantages of SDT, so finding suitable sonosensitizers is still the key to enhancing SDT efficiency. MOF-based sonosensitizers largely improve the SDT efficiency, compared to the MOFs’ applications in gas adsorption and storage as well as catalysis, the number of MOF-based sonosensitizers that can be used in SDT in the medical field is still very small, and there is a need to explore better MOF-based sonosensitizers to adapt to SDT as a new type of treatment modality. As micro- and nanomaterials, the particle size of MOFs affects the transport efficiency of sonosensitizers. The nanoscale size of MOFs allows them to be passively targeted at tumor sites through enhanced permeability and retention effect (EPR). Still, too-large particles tend to cause high accumulation of sonosensitizers in vivo. They cannot be excreted, too small particles affect the loading rate of sonosensitizers, and it is challenging to synthesize ideal MOFs rapidly and reproducibly. MOFs may also face a series of problems in vivo, such as whether they are degradable, have better biocompatibility, and maintain safe and stable performance. In addition, most current studies are based on the short-term toxicity of MOFs. In order to more comprehensively evaluate the impact of MOFs on the body, the problems caused by the long-term toxicity of MOFs cannot be ignored. Therefore, on the one hand, more efficient and highly biocompatible micro/nanoparticles should be screened, and the diversity of MOFs is a potential advantage in this regard with high plasticity. At the same time, a similar selection of suitable particle size for different cancers will bring unexpected results. In order to better expand the prospects of sonosensitizers in combination with SDT in cancer, the long-term toxicity of sonosensitizers itself should be treated with caution.

On the other hand, the research on the mechanism of action of SDT is still in its infancy, there are no studies to confirm the exact mechanism of SDT treatment of tumors, and there are still major challenges in the clinical research of cancer treatment. A more comprehensive mechanism of action of SDT should continue to be pursued to better achieve the effect of precision therapy. At present, cancer treatment has gradually developed from single surgery and drug therapy to multi-drug or combination therapy, which can better avoid poor treatment effects and drug resistance. SDT-based combination therapy will also be one of the future development trends, such as SDT and traditional chemotherapy synergistic treatment, SDT and CDT synergistic treatment. SDT can also be a synergistic treatment with PDT, because most sonosensitizers still have the role of photosensitizers, which can improve treatment for tumors. In summary, the rapidly developing micro- and nanotechnology in the medical field also still faces many challenges. SDT based on MOF-based sonosensitizer is still in the beginning stages, and many problems remain to be solved. The rational selection of MOF-based sonosensitizers may become the key to the practical application of SDT, which will promote the development of SDT in tumor therapy.

## Data Availability

Not applicable.
